# Diagnosis of acute cystitis in primary care: symptom-based versus urinalysis-based diagnosis

**DOI:** 10.1017/S1463423622000627

**Published:** 2022-11-17

**Authors:** Rian Lelie- van der Zande, Ellen S. Koster, Marion Grol, Kurt G. Naber, Jakhongir F. Alidjanov, Martina Teichert, Marcel L. Bouvy

**Affiliations:** 1 Department of Pharmacoepidemiology and Clinical Pharmacology, Utrecht Institute for Pharmaceutical Sciences, Utrecht University, Utrecht, the Netherlands; 2 General Practice Vreeswijk, Nieuwegein, the Netherlands; 3 Department of Urology, Technical University of Munich, Munich, Germany; 4 Department of Urology, Pediatric Urology and Andrology, Justus-Liebig University of Giessen, Giessen, Germany; 5 Department of Clinical Pharmacy & Toxicology, Leiden University Medical Center, Leiden, the Netherlands

**Keywords:** diagnosis, GP, primary care, questionnaire, urinary tract infection, urinalysis

## Abstract

**Aim:**

This study aimed to provide insight into the congruity of acute cystitis (AC) diagnosis in women, measured both by the Acute Cystitis Symptom Score (ACSS) questionnaire and urine test(s).

**Background:**

The ACSS questionnaire was developed as a self-administering tool for assessing urinary symptoms, quality of life (QoL) and treatment outcomes in healthy, nonpregnant female patients.

**Methods:**

This prospective observational cohort study compared AC diagnosis based on the questionnaire with a GP diagnosis based on dipstick/dipslide test(s). ACSS questionnaire form A (typical and differential symptoms, QoL and relevant conditions) was filled in by the patient group, women suspected for AC visiting a GP practice with a urine sample, and the reference group, women visiting a community pharmacy for any medication. Analyses were performed assuming that the GP diagnosis based on urine test(s) was correct. Divergent result(s) of urine test(s) and ACSS questionnaire were analysed for scores of all individual questionnaire domains. Statistical analyses included descriptive statistics and the positive predictive value (PPV) and the negative predictive value (NPV) of the ACSS questionnaire and the urine test(s).

**Findings:**

In the patient group, 59 women were included, 38 of whom a GP positively diagnosed for AC. The reference group included 70 women. The PPV of the ACSS questionnaire was 77.3%, and the NPV was 73.3%. Analysis of patient data for divergent results showed that differential symptoms, QoL and relevant conditions explained false-positive and false-negative results. Revised results (most probable diagnosis) based on this analysis showed a PPV and NPV of 88.6% and 73.3% for the ACSS questionnaire and 100% and 76.2% for the urine test(s). For use in primary care, a reduction in false-positive and false-negative results can be achieved by including scores for differential symptoms, QoL and relevant conditions, alongside a total typical symptoms score of 6 or higher.

## Introduction

In healthy, nonpregnant women, acute cystitis (AC) is one of the most frequent indications for antibiotic prescriptions in primary care (Foxman, 2002; Lelie-van der Zande *et al*., 2020; Lelie-van der Zande *et al*., 2021a). A correct diagnosis is vital to minimise unnecessary antibiotic prescriptions since the overuse of antibiotics is recognised as the main driver of antimicrobial resistance (WHO, 2020). Nevertheless, the undertreatment of an AC episode may negatively impact women’s quality of life (QoL) (Renard *et al*., 2015; Boeri *et al*., 2017; Wagenlehner *et al*., 2018; Ennis *et al*., 2018; Medina and Castillo-Pino, 2019).

The most reliable diagnosis is made by performing a urine culture, which is expensive and time-consuming and therefore less suitable for everyday practice. Alternatively, the nitrite dipstick test (fast but may give a false-negative result) (NHG, 2020), the urinary sediment (requires microscopy and a skilled investigator) and the dipslide (has a delay of 24 h for the results) are most widely applied (Knottnerus and Geerlings, 2013). In the Netherlands, the GP guidelines recommend performing a dipstick test followed by a dipslide test or culture if necessary (Bouma *et al*., 2019).

The high incidence of recurring AC in women leads to frequent GP visits (Meijer *et al*., 2021). Shortages of health care providers (van der Horst and de Wit, 2020) and diagnoses at out-of-hours primary care where antibiotics are prescribed without urine test(s) (Spek *et al*., 2020) urge considering efficient ways to deal with recurring AC. Many women may recognise an AC episode themselves, potentially based on previous experiences (Lelie-van der Zande *et al*., 2021b).

The Acute Cystitis Symptom Score (ACSS) questionnaire was developed as an instrument to support self-reported AC diagnosis in female patients (Alidjanov *et al*., 2014). The questionnaire assesses the severity of typical and differential symptoms and their impact on QoL in women with suspected AC to differentiate it from other urological disorders while signalling differential conditions and assessing patient-reported outcomes (Alidjanov *et al*., 2013; 2014; 2016; 2018a; 2018b; 2019; 2020a; 2020c). Previously, the questionnaire was clinically validated in patients with suspected AC in hospital settings (Alidjanov *et al*., 2014; 2015; Magyar *et al*., 2018; Di Vico *et al*., 2020), whereas AC patients in the Netherlands turn to primary care for diagnosis and treatment.

This study aimed to provide insight into the congruity of AC diagnosis, measured by the ACSS questionnaire and urine test(s), after translation and linguistic assessment of the questionnaire in the Dutch language.

## Materials and methods

### Study design

**Figure 1.**
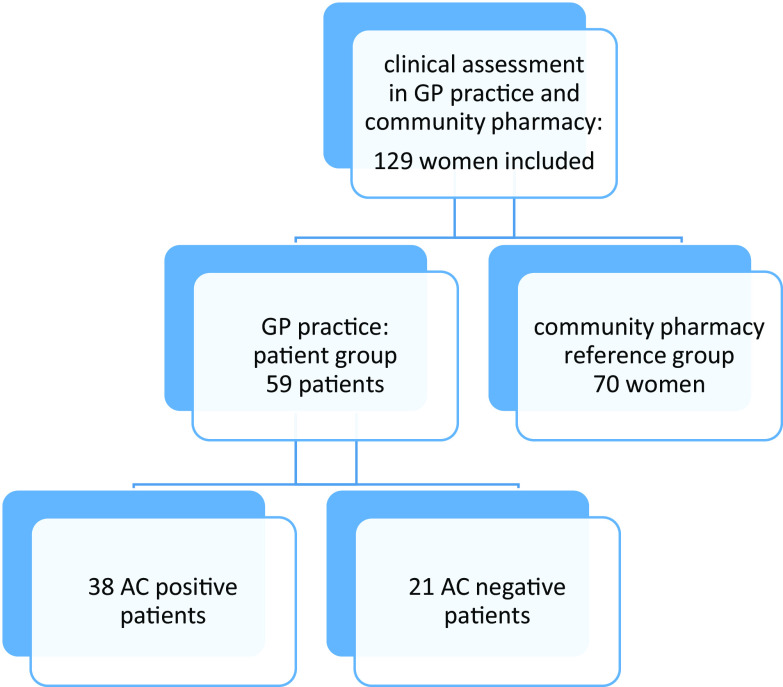
Study flowchart

In this prospective observational cohort study, AC diagnosis based on the self-reporting ACSS questionnaire was compared to AC diagnosis based on urine test(s) because of AC symptoms (Bouma *et al*., 2019). According to the actual GP procedure, AC-positive patients were defined as patients with a positive dipstick or a positive dipslide in the case of a negative dipstick (Figure 1). The study was performed between February 2020 and July 2021, with an interruption from mid-March until October 2021 because of the COVID-19 pandemic.

### Settings and participants

The study was performed in two GP practices in different locations. In the first location, a community pharmacy and a GP practice (five part-time working GPs) were situated in a rural area, and they cooperated in a regional primary care network. In the second location, both the pharmacy and the GP practice (four GPs) were located in an urban health centre and closely collaborated. In both GP practices, the practice assistants were responsible for performing urine tests and preparation of antibiotic prescriptions. Antibiotic prescriptions were approved by the GP before transferring them to the community pharmacy.

Women who gave a urine sample because of AC symptoms received information about the study procedures and were invited to participate in the patient group. Patient permission was solicited to obtain urine test result(s) from the GPs. Women visiting the GP practice were excluded from the patient group if (1) they were not fluent in Dutch, (2) had antimicrobial therapy within two weeks prior to the GP visit, (3) were pregnant, (4) used other drug therapy (such as NSAIDs) within 48 h prior to the GP visit that might affect the severity of symptoms, (5) had recent bladder catheterisation or other invasive manipulations in the urinary tract or (6) had known anatomic or functional abnormality of the urinary tract. Women who visited one of the community pharmacies for any prescription except an antibiotic or painkiller and without suspected AC at the time of inclusion received information about study procedures and were invited to participate in the reference group (Figure 1).

### The acute cystitis symptom score questionnaire

#### Questionnaire validation studies and content

The ACSS questionnaire consists of forms A and B. Form A is used at the start of symptoms and contains four domains: [1] the typical domain with six questions about AC symptoms, [2] the differential domain with four questions about differential symptoms, [3] the QoL domain with three questions and [4] the relevant condition domain with questions on menses, pregnancy, premenstrual syndrome, menopause and diabetes mellitus. Symptoms in the first two domains are scored from 0 (not present) to 3 (severe) to measure symptom severity. QoL items are scored from 0 (not affected) to 3 (extremely affected). Items of the relevant condition domain have binary yes/no answer options.

Follow-up form B is used to assess the therapy result (Alidjanov *et al*., 2016). Form B contains all form A domains and an additional domain on dynamics: change of symptoms approximately one week after the first GP visit, scored as 0 (feel back to normal with all symptoms gone), 1 (feel much better with most symptoms gone), 2 (feel somewhat better with most symptoms still present), 3 (feel about the same with no changes in symptoms) and 4 (feel worse with a worsened condition). Previous validation studies have shown an optimal threshold for the total typical symptoms score of 6 for diagnosing AC (Alidjanov *et al*., 2014; 2015; Magyar *et al*., 2018; Di Vico *et al*., 2020).

#### Translation, linguistic and cognitive assessment

The translation and linguistic assessment of the Dutch version of the ACSS questionnaire were performed per the Linguistic Validation Manual for Patient-Reported Outcomes instrument guidelines (Acquadro *et al*., 2014). Two independent translators produced primary, forward translations into the Dutch language from the validated Russian and American English ACSS versions, respectively (Alidjanov *et al*., 2020b). These translations were discussed with three women who experienced AC in the past, a GP and a community pharmacist, leading to the first version, which was back-translated into English by an independent native speaker and compared with the original English version to detect relevant differences.

The resulting second version was used for cognitive assessment carried out with nine Dutch-speaking women who had varying educational levels and ages and did or did not experience AC in the past. The researchers discussed comments from these women with the developers of the original ACSS questionnaire. This discussion led to the final study version of the Dutch ACSS questionnaire (Supplementary Figure 1).

### Data collection

Women in the patient group filled in the Dutch ACSS questionnaire form A manually, electronically or telephonically, identifying essential characteristics (e.g., age and educational level). Afterwards, the researcher made an appointment with the patient to fill in form B telephonically after finishing treatment. Only patients who filled in questionnaire form A within four days after visiting the GP practice were included. Notably, reference group women filled in form A with essential characteristics when visiting the community pharmacy.

### Definition of outcomes: Diagnosis

The GP practices performed a dipstick test for all patients suspected for AC, assessing the presence of nitrite and leukocytes (5–10 white blood cells per ml) (NHG, 2020). If nitrite was positive, an antibiotic was prescribed according to the urinary tract infections (UTI) guideline (Bouma *et al*., 2019); if nitrite was negative and leukocytes were positive a dipslide was performed (Bouma *et al*., 2019). Moreover, according to the guideline, a dipslide was performed if leukocytes were negative, but AC was suspected based on patient complaints. An antibiotic was prescribed if the dipslide was positive (colony-forming units ≥ 10^4^/ml). AC-positive patients were defined as patients with one or more AC symptoms (dysuria, frequency, urgency, incomplete bladder emptying, pain in lower abdomen or haematuria) and a positive dipstick or positive dipslide.

### Data analysis

Descriptive statistics were used for demographic characteristics. Education levels were assigned according to the Statistic Netherlands database (CBS, 2020). Comparable to earlier validation studies, the reliability of the translated ACSS questionnaire was analysed by calculating Cronbach’s α and Guttman split-half reliability for the total questionnaire and all individual domains. Cronbach’s α was also calculated for the typical symptoms domain combined with the QoL domains, with only three items. Moreover, because of higher reliability, splitting into halves was performed depending on odd and even ordinary numbers of items. For the calculation of Cronbach’s α, no missing values were allowed. Therefore, differential question 10a of the ACSS (measurement of body temperature) was marked as ‘temperature < 37.5 °C’ if a woman had not measured her temperature but had answered question 10 with ‘having no feeling of fever’.

The translated ACSS questionnaire was considered reliable if Cronbach’s α and Guttman split-half reliability were higher than 0.80. For discriminative ability, receiver operating characteristics (ROC) analysis using a non-parametric test was performed on the total typical symptoms score for AC-positive patients as a tested variable to determine the best cut-off value. Typical symptom scores, differential symptom scores, QoL item scores and domain scores were compared for AC-positive patients and AC-negative patients, for AC-positive patients and reference group and for AC-negative and reference group, using Wilcoxon signed ranks test (significant if *P < 0.05)*. These analyses were performed assuming that a GP diagnosis with urine test(s) was correct. Furthermore, a Mann-Whitney U test was performed to compare scores for individual questionnaire items for AC-positive patients, AC-negative patients and reference group women (significant if *P < 0.05*). Moreover, a pairwise Wilcoxon signed rank test was performed to compare scores of AC-positive patients before and after antibiotic treatment (significant if *P < 0.05*).

The positive and negative results of the ACSS questionnaire based on the total typical symptoms score (> 6 or < 6) and the GP guideline diagnosis based on the urine test(s) were compared. For patients with a divergent diagnosis for the ACSS questionnaire and GP practice with typical symptom scores, the scores for differential symptoms, QoL and additional data were mapped and discussed with the research team GP to determine the most probable diagnosis. ROC analysis was also performed for the most probable diagnosis with AC-positive patients and reference group women plus AC-negative patients (stated variable) and the total typical symptoms score (tested variable). Moreover, typical symptom scores, differential symptom scores, QoL item scores and domain scores were compared for AC-positive patients and AC-negative patients, for AC-positive patients and reference group, and for AC-negative patients and reference group using Mann-Whitney U test (significant if *P < 0.05*). Data were analysed using Statistical Package for Social Science (IBM SPSS) for Windows, version 27.0. Additionally, R v.3.5.2 with in-built and additional packages was used for comparative analyses and graphical representation of the results (R Development Core Team, 2016; Heike *et al*., 2017).

## Results

### Participants

In total, 64 patients completed the ACSS questionnaire. Five patients were excluded because questionnaire A was completed more than four days after the urine test(s) in GP practice. Of the remaining 59 patients, 38 were AC-positive patients, and 35 could be followed up on treatment results. The AC-negative patients were not treated and had no follow-up.

A total of 70 women were included in the reference group (Figure 1). No statistically significant differences were found between AC-positive patients and reference group women in age and educational level (Table 1). For all patients, the median number of days between the start of symptoms and a GP visit was 4.0 days (0–30) and 74.5% of all patients visited a GP practice within five days after symptoms started.

**Table 1.** Participants’ characteristics. AC diagnosis according to GP guideline

	Patient group (n = 59)		Reference group (n = 70)
	AC-positive patients (n = 38)	AC-negative patients (n = 21)	Reference group (n = 70)
Median age in years (min-max)	51.5 (19–80)	48.0 (27–75)	54.5 (19–79)
Education, number (percentage)			
Primary	34.2% (13)	33.3%) (7)	30.0% (21)
Secondary	39.5% (15)	33.3%) (7)	34.3% (24)
Higher	26.3% (10)	33.3% (7)	35.7% (25)

### Reliability of the ACSS questionnaire

Cronbach’s α and Guttman split-half reliability for the total questionnaire, for the typical domain and the QoL plus typical domain were higher than 0.80 (Table 2). Cronbach’s α for the differential domain containing severely divergent items was 0.49. ROC analysis performed for a GP diagnosis with AC-positive patients and reference group women plus AC-negative patients (stated variable) and the total typical symptoms score (tested variable) resulted in an area under the curve (AUC) of 0.93 (95%-CI, 0.89–0.98), with a sensitivity of 88.5% and specificity of 89.0% at a cut-off point of 6 for the summary score of the typical domain in the ACSS.

**Table 2.** Reliability analysis per domain for all included patients and controls

Domain	All respondents (n = 129)
Total questionnaire^ª^	
Cronbach’s alpha (95%-CI)	0.87 (0.84–0.90)
Guttman split-half coefficient	0.85
Typical domain	
Cronbach’s alpha (95%-CI)	0.82 (0.77–0.87)
Guttman split-half coefficient	0.81
Differential domain	
Cronbach’s alpha (95%-CI)	0.49 (0.33–0.62)
Quality of Life domain	
Cronbach’s alpha (95%-CI)	0.87 (0.82–0.90)
Quality of Life + Typical domain	
Cronbach’s alpha (95%-CI)	0.90 (0.87–0.92)
Guttman split-half coefficient	0.83

ª Typical, differential and quality of life domains. As the items of the additional domain are dichotomous, they were not included in this analysis.

### Scores for typical symptoms, differential symptoms and quality of life

**Figure 2.**
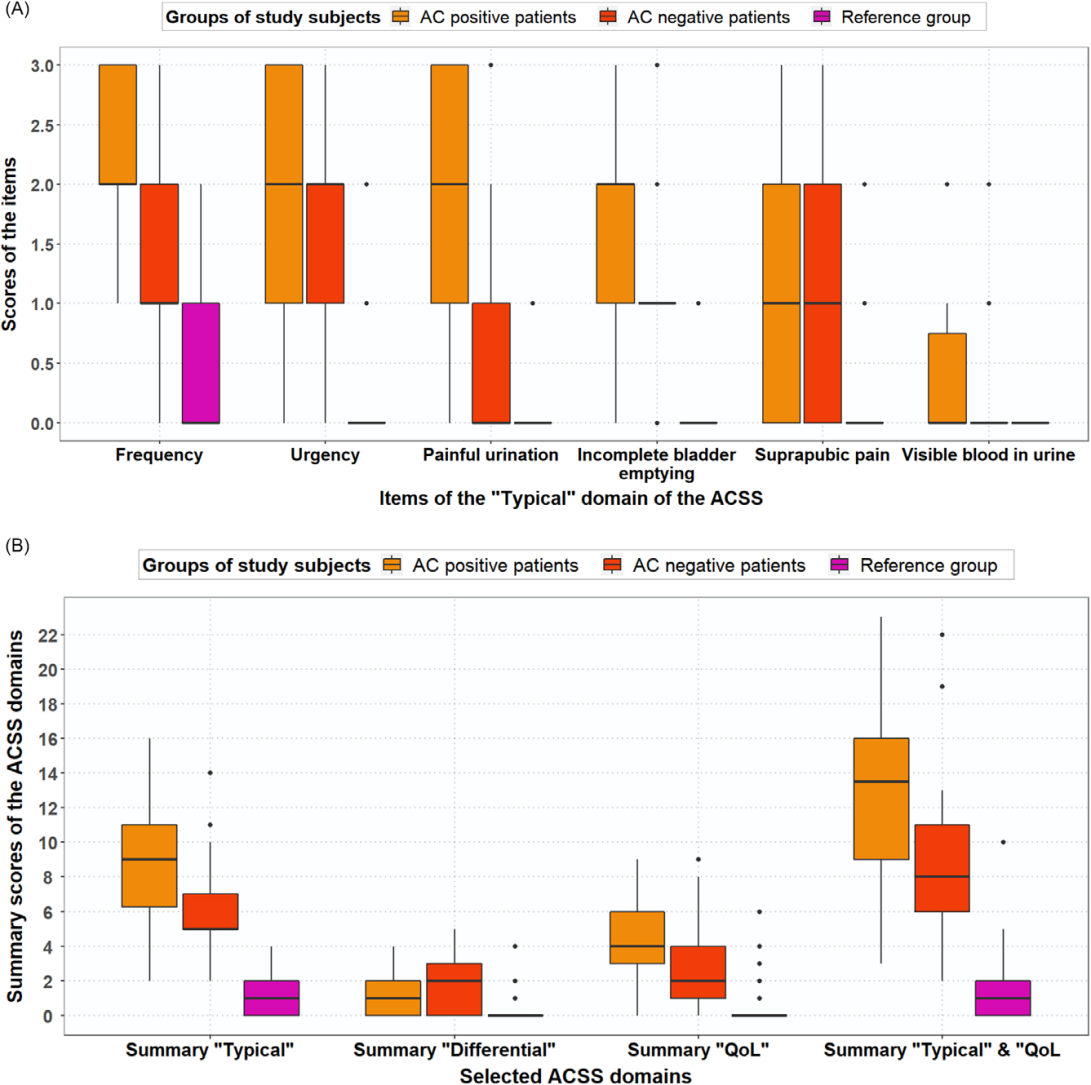
(A) Boxplot with typical symptom scores (median, IQR) for AC-positive patients (n = 38), AC-negative patients (n = 21) and reference group (n = 70), based on GP guideline diagnosis. (B) Boxplot with ACSS domain scores (median, IQR), both for AC-positive patients (n = 38), AC-negative patients (n = 21) and reference group (n = 70), based on GP guideline diagnosis

Scores for frequent urination of small volumes, urgent urination, feeling pain or burning when passing urine, incomplete bladder emptying, total typical domain, vaginal discharge, general discomfort and total QoL domain differed significantly for AC-positive patients and AC-negative patients (Table 3). The median (mean) typical symptom scores for frequency were 2.0 (2.13), 1.5 (1.43) and 1.0 (0.5) for AC-positive, AC-negative and reference group women, respectively (Figure 2A). Summary scores of the domains (Typical, Differential, Qol) for AC-positive patients versus reference group and AC-negative patients versus reference group differed significantly (Table 3, Figure 2B).

**Table 3.** Comparison of typical symptom scores, differential symptom scores, QoL item scores and domain scores between a) AC-positive patients (n = 38) versus AC-negative patients (N = 21); B) AC-positive patients (n = 38) versus reference group (n = 70); C) AC-negative patients (n = 21) versus reference group (n = 70). AC diagnosis according to GP guideline; differences statistically significant if *P* < 0.05

		p-values (significant *P* < 0.05)		
Compared groups		A	B	C
Typical domain	Frequency	0.013	<0.001	<0.001
	Urgency	0.037	<0.001	<0.001
	Painful urination (dysuria)	0.009	<0.001	<0.001
	Incomplete bladder emptying	0.021	<0.001	<0.001
	Suprapubic pain	0.592	<0.001	<0.001
	Visible blood in urine	0.925	<0.001	<0.001
Differential domain	Flank pain	0.570	<0.001	<0.001
	Vaginal discharge	0.006	0.090	<0.001
	Urethral discharge	0.372	0.193	0.027
	High body temperature, fever	0.403	0.016	0.378
Quality of life (QoL) domain	General discomfort	0.020	<0.001	<0.001
	Everyday activities/ work	0.059	<0.001	<0.001
	Social activities	0.328	<0.001	0.005
Domains	Typical	0.003	<0.001	<0.001
	Differential	0.151	<0.001	<0.001
	QoL	0.031	<0.001	<0.001
	Typical plus QoL	0.002	<0.001	<0.001

**Figure 3.**
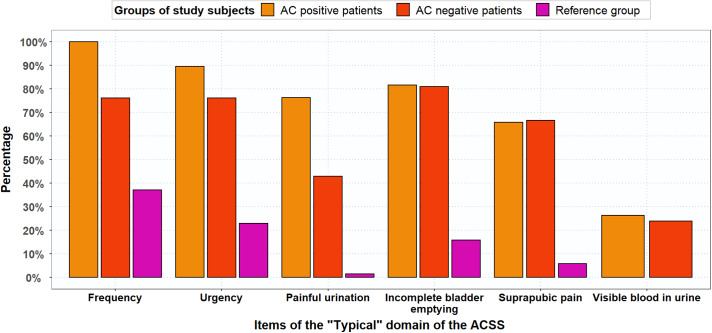
Percentages of AC-positive patients (n = 38), AC-negative patients (n = 21) and reference group (n = 70) for typical symptoms with score ≥1 (mild, moderate or severe symptoms). based on GP guideline diagnosis

Figure 3 shows that both AC-positive and AC-negative patients experienced AC symptoms. The percentages of AC-positive patients and reference group women that experienced frequent urination were 100% and 37.1%. Of the AC-positive patients, 100% scored mild, moderate or severe for frequency (5–6 times or more daily), whereas this percentage was 37% for the reference group women (Figure 3): 24% scored mild and 13% scored moderate for frequency.

Vaginal and urethral discharge scores did not differ significantly for AC-positive patients and reference group women contrary to scores for individual typical domain symptoms, remaining differential symptoms, QoL domain items and total symptom scores for all domains, which were significantly higher for AC-positive patients than for reference group women (*P* < 0.05) (Table 3). Moreover, the median (mean) typical symptom score for blood in the urine was lowest at 0.00 (0.32) for AC-positive patients and comparable to scores for blood in the urine for AC-negative patients. For AC-positive patients, mean QoL scores were 2.00 for general discomfort, 1.53 for influence on work/daily activities and 0.79 for influence on social activities.

### Comparison of ACSS questionnaire result and GP diagnosis

In the GP practices, 38 patients were diagnosed as AC-positive and 21 as AC-negative. Based on the total typical symptoms score ≥ 6, the ACSS questionnaire diagnosed 44 patients as AC-positive and 15 as AC-negative. The positive predictive value (PPV) of the ACSS questionnaire (total typical symptoms score ≥ 6) was 77.3%, whereas the negative predictive value (NPV) was 73.3%.

#### Analysis of divergent diagnoses

Based on an ACSS questionnaire score < 6, four patients did not have AC, while the urine test(s) showed a positive result (Table 4). In three cases, the patient probably rated the symptom severity too low. When filling in the questionnaire, one patient reported she always recognised having an AC episode by urine scent and goosebumps when urinating. The conclusion was that the ACSS questionnaire probably was false-negative for AC for these four cases.

**Table 4.** Patients with negative ACSS questionnaire result and positive urinary test(s) result but most probable diagnosis AC-positive (false-negative ACSS)

Patient number	Age (yrs)	ACSS form A result^a^	Total score typical symptoms^b,^ ^f^	Total score differential symptoms^d,^ ^f^	Total score QoL^e,^ ^f^	ACSS form B result^g^	Urinary test(s) result^h^	most probable diagnosis^k^	Argumentation for ACSS false-negative
003	70	0	4	0	5	0	1	1	Clear growth dipslide (10^7), scores QoL discomfort and daily activities moderate; probably symptoms rated too low
016	23	0	5	2	1	1	1	1	Moderate scores for frequency, abdominal pain, flank pain, mild for incomplete emptying of the bladder; probably symptoms rated too low.
041	47	0	3	2	3	2	1	1	Clear growth dipslide (10^5), ACSS form B: most symptoms still present; probably symptoms rated too low
043	63	0	2	1	1	1	1	1	Patient recognises AUC by urine scent and goosebumps from bladder to head when urinating

a Result ACSS questionnaire based on total typical symptoms score ≥ 6:1 = AC-positive; 0 = AC-negative.

b Total typical symptoms score: frequency + urgency + burning pain when urinating + incomplete emptying of the bladder + abdominal pain + blood seen in urine.

d Total differential symptoms score: loin pain + vaginal discharge + urethral discharge + high body temperature + temperature specified.

e Total QoL score: general discomfort + influence on work/ daily activities + influence on social activities.

f severity score per symptom/ item: 0 = absent, 1 = mild, 2 = moderate, 3 = severe.

g ACSS B: treatment result: 0 = all symptoms have gone away; 1 = majority of symptoms have gone away; 2 = majority of symptoms is still present; 3 = no changes in my symptoms; 4 = my condition is worse.

h Urinary test result: dipstick and eventual dipslide: 1 = AC-positive; 0 = AC-negative.

k most probable diagnosis, based on evaluation with independent GP: 1 = AC-positive; 0 = AC-negative.

The ACSS questionnaire diagnosed ten patients as AC-positive, while the urine test(s) showed a negative result. In five cases, differential diagnostics were probably indicated because of differential symptoms and blood loss that worried the patient. In one of these patients, the GP had already started differential diagnostics. The conclusion was that the ACSS questionnaire probably was false-positive for AC (Table 5) but was a good indicator to perform differential diagnostics.

**Table 5.** Patients with positive ACSS questionnaire result and negative urinary test(s) result but most probable diagnosis AC-negative (false-positive ACSS)

Patient number	Age (yrs)	ACSS form A result^a^	Total score typical symptoms^b,^ ^f^	Total score differential symptoms^d,^ ^f^	Total score QoL^e,^ ^f^	ACSS form B result^g^	Urinary test(s) result^h^	most probable diagnosis^k^	Argumentation for false-positive ACSS
004	27	1	6	2	2	2	0	0	GP started differential diagnostics because of patient complaining of irritable bowel, vaginal discharge, obstipation
006	62	1	8	6	1	2	0	0	Form A: moderate scores for flank pain/ vaginal discharge/fever; Form B: majority of symptoms still present; postmenopausal; possibly vaginal atrophy; differential diagnostics indicated
014	49	1	7	3	3	1	0	0	Forms A and B: mild vaginal/ urethral discharge; score QoL discomfort severe; Form B: majority of typical symptoms gone away; possibly vaginal bacteriosis; differential diagnostics indicated
021	29	1	10	3	9	–	0	0	Severe complaints for frequency, urgency, abdominal pain and flank pain which could indicate a higher UTI, but then a positive urine test would be expected. QoL severely affected. Differential diagnostics indicated.
045	60	1	7	0	2	1	0	0	Moderate scores for frequency, urgency, blood in urine (patient was very worried about it), mild score for burning pain when urinating; possibly vaginal atrophy; differential diagnostics indicated.

a Result ACSS questionnaire based on total typical symptoms score ≥ 6:1 = AC-positive; 0 = AC-negative.

b Total typical symptoms score: frequency + urgency + burning pain when urinating + incomplete emptying of the bladder + abdominal pain + blood seen in urine.

d Total differential symptoms score: loin pain + vaginal discharge + urethral discharge + high body temperature + temperature specified.

e Total QoL score: general discomfort + influence on work/ daily activities + influence on social activities.

f severity score per symptom/ item: 0 = absent, 1 = mild, 2 = moderate, 3 = severe.

g ACSS B: treatment result: 0 = all symptoms have gone away; 1 = majority of symptoms have gone away; 2 = majority of symptoms is still present; 3 = no changes in my symptoms; 4 = my condition is worse.

h Urinary test result: dipstick and eventual dipslide: 1 = AC-positive; 0 = AC-negative.

k most probable diagnosis, based on evaluation with independent GP: 1 = AC-positive; 0 = AC-negative.

The other five patients with an AC-positive diagnosis of the questionnaire and a negative urine test had evident AC symptoms, but in four of them, the urine test(s) most probably was negative because of a short duration of stay of urine in the bladder. The fifth patient forgot nitrofurantoin prophylaxis after sexual intercourse but ingested 200 mg of nitrofurantoin before visiting GP practice. In one of the four patients, the symptoms subsided after using an extended prescription of fosfomycin from an earlier AC episode, which she had at home. Despite negative urine test(s), the GP prescribed one patient nitrofurantoin because of symptoms. The conclusion was that the GP practice diagnosis probably was false-negative for AC (Table 6).

**Table 6.** Patients with positive ACSS questionnaire result and negative urinary test(s) result but most probable diagnosis AC-positive (false-negative result for GP)

Patient number	Age (yrs)	ACSS form A result^a^	Total score typical symptoms^b,^ ^f^	Total score differential symptoms^d,^ ^f^	Total score QoL^e,^ ^f^	ACSS form B result^g^	Urinary Test(s) result^h^	most probable diagnosis^k^	Argumentation for most probable diagnosis
002	39	1	10	3	1	1	0	1	Evident AUC symptoms; symptoms disappeared by natural course; no vaginal and urethral discharge in ACSS B; urine test negative, possibly by short urine duration of stay in bladder.
011	53	1	11	1	2	1	0	1	Evident AUC symptoms, score QoL discomfort moderate; patient used delayed prescription fosfomycin (from earlier AUC) at home after negative urine test, after which frequency and burning pain disappeared; possibly short urine duration of stay in bladder.
025	49	1	7	1	1	0	0	1	Evident AUC symptoms; symptoms disappeared by natural course; urine test negative possibly by short duration of stay in bladder.
033	48	1	14	0	8	0	0	1	Evident AUC symptoms, scores QoL discomfort and work severe; patient forgot prophylaxis after sexual intercourse but ingested 200 mg nitrofurantoin before visiting the GP.
048	73	1	7	0	4	0	0	1	Evident AUC symptoms; moderate scores for QoL discomfort and daily activities; GP prescribed antibiotic based on symptoms, urine test negative possibly by short duration of stay in bladder.

a Result ACSS questionnaire based on total typical symptoms score ≥ 6:1 = AC-positive; 0 = AC-negative.

b Total typical symptoms score: frequency + urgency + burning pain when urinating + incomplete emptying of the bladder + abdominal pain + blood seen in urine.

d Total differential symptoms score: loin pain + vaginal discharge + urethral discharge + high body temperature + temperature specified.

e Total QoL score: general discomfort + influence on work/ daily activities + influence on social activities.

f severity score per symptom/ item: 0 = absent, 1 = mild, 2 = moderate, 3 = severe.

g ACSS B: treatment result: 0 = all symptoms have gone away; 1 = majority of symptoms have gone away; 2 = majority of symptoms is still present; 3 = no changes in my symptoms; 4 = my condition is worse.

h Urinary test result: dipstick and eventual dipslide: 1 = AC-positive; 0 = AC-negative.

k most probable diagnosis, based on evaluation with independent GP: 1 = AC-positive; 0 = AC-negative.

#### Revision of PPV, NPV and ROC based on analysis of divergent results

**Figure 4.**
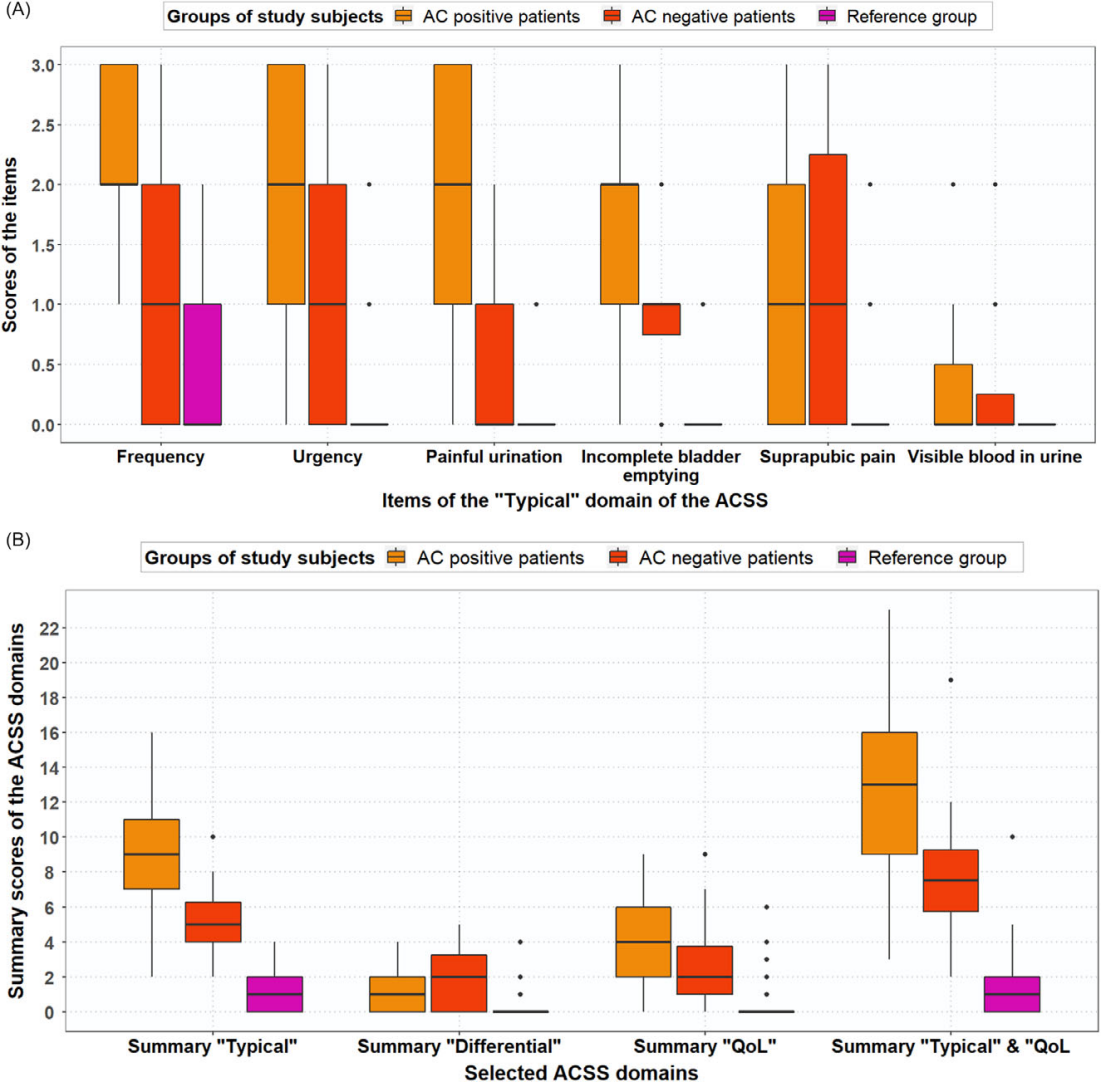
(A) Boxplot with typical symptom scores (median, IQR) for AC-positive patients (n = 43), AC-negative patients (n = 16) and reference group (n = 70), based on most probable diagnosis, after evaluation of divergent results of ACSS questionnaire and urine test(s). (B) Boxplot with ACSS domain scores (median, IQR), both for AC-positive patients (n = 43), AC-negative patients (n = 16) and reference group (n = 70), based on most probable diagnosis, after evaluation of divergent results of ACSS questionnaire and urine test(s)

**Figure 5.**
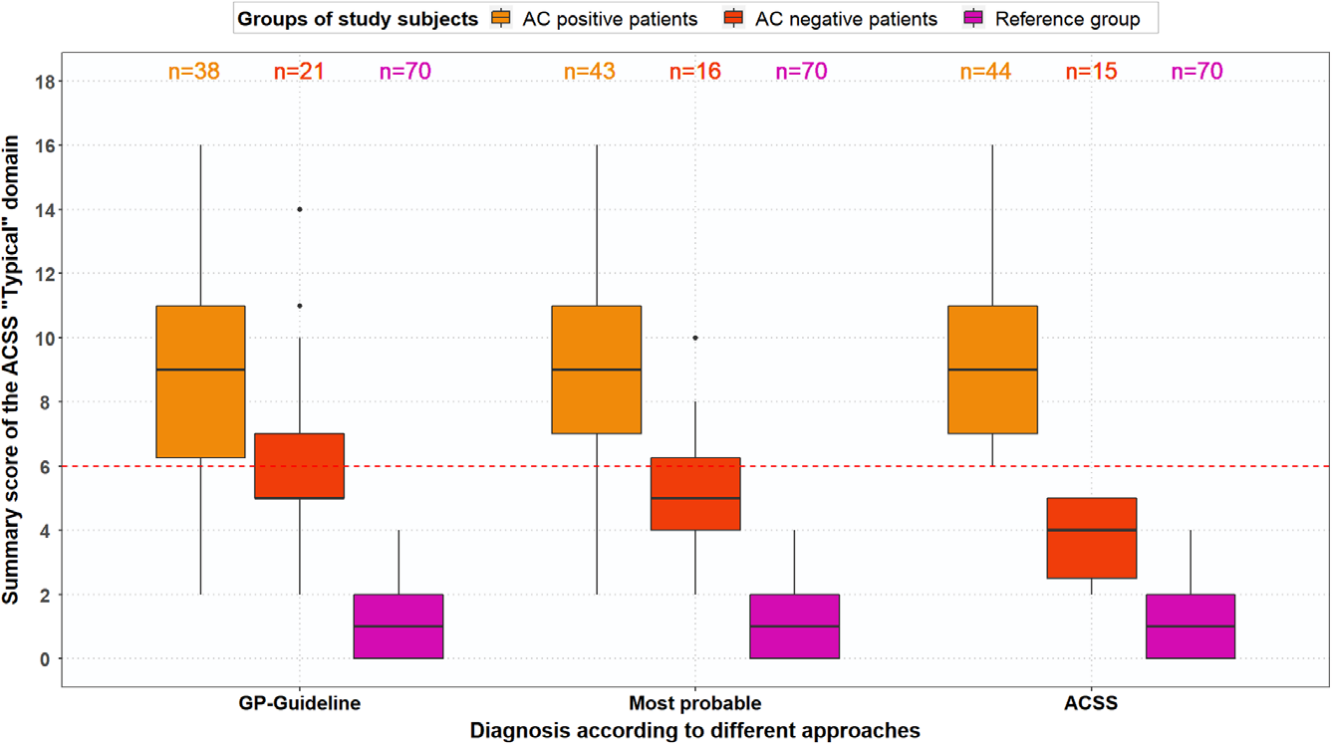
Boxplot with total typical symptom scores (median, IQR) for AC-positive patients n = 38), AC-negative patients (n = 21) and reference group (n = 70), (a) based on GP guideline diagnosis, (b) based on most probable diagnosis after evaluation of divergent results of ACSS questionnaire and urine test(s), and (c) based on ACSS diagnosis (summary score ≥6)

The PPV and NPV were recalculated for the ACSS questionnaire and GP diagnosis based on the most probable diagnosis. Figures 4A and 4B show boxplots for the typical symptoms and the domains for AC-positive and AC-negative patients and the reference group based on the most probable AC diagnoses. The ACSS questionnaire resulted in a PPV of 88.6% and an NPV of 73.3%, while the revised GP diagnosis resulted in a PPV of 100% and an NPV of 76.2%. Moreover, ROC analysis for most probable diagnosis with AC-positive patients and reference group women plus AC-negative patients (stated variable) and the total typical symptoms score (tested variable) resulted in an AUC of 0.97 (95%-CI, 0.94–0.99) with a sensitivity of 90.7% and specificity of 94.2% at a cut-off point of 6. Figure 5 shows that the total typical symptoms score for the most probable diagnosis has a clearer distinction between AC-positive and AC-negative patients in comparison to the summary scores achieved by using the GP guideline approach.

### Comparison of domain scores ACSS forms A and B

For 35 of 38 (92%) patients who received antibiotic treatment after being diagnosed as AC-positive at a GP practice, ACSS questionnaire form B was available. The median interval between filling in questionnaire forms A and B was ten days (6–35). A comparison of symptom and item scores of the typical, differential and QoL domains for AC-positive patients before and after antibiotic treatment showed significantly lower scores after treatment (*P* < 0.05) than at the start of treatment for all symptoms and items, except abnormal vaginal and urethral discharge and specified body temperature, with the caveat that only three patients had taken their temperature. When filling in questionnaire form B, 54.3% (*n* = 19) of the AC-positive patients who received antibiotic treatment felt back to normal, while 29.0% (*n* = 11) felt much better.

## Discussion

The reliability, sensitivity and specificity of the Dutch translation of the ACSS questionnaire were comparable to results of earlier ACSS validation studies. However, assuming that GP urine test result(s) were correct, the PPV and NPV for the questionnaire were approximately 25% lower than those for the urine test(s). Divergent results for the ACSS questionnaire and urine test(s) probably were caused by [1] patients (ACSS symptom score < 6) rating symptom severity too low or recognising an AC episode by urine scent (false-negative ACSS), [2] symptoms caused by a differential condition or a high score on blood in the urine (false-positive ACSS) and [3] short duration of stay of urine in the bladder (false-negative GP diagnosis).

The Dutch translation of the ACSS questionnaire showed a sensitivity of 88.5% and specificity of 89.0% based on a correct GP guideline diagnosis, which increased to 90.7% for sensitivity and 94.2% for specificity based on a revised, most probable diagnosis at the cut-off point of ≥ 6 for the total typical symptoms score, which complies with earlier ACSS validation studies (Alidjanov *et al*., 2014; Magyar *et al*., 2018; Di Vico *et al*., 2020).

For practical use in primary care, a reduction in false-positive and false-negative results is possible, achieved by including scores for differential symptoms, QoL scores, relevant conditions and moderate or severe scores for blood in urine in the evaluation of the ACSS questionnaire in patients with a typical symptom score of 6 or higher.

A false-negative ACSS may result from patients rating symptoms too low, possibly because the questionnaire could not be filled in when handing in the urine at a GP practice. Moreover, when filling in the questionnaire up to 4 days after the GP visit, some patients may have struggled to recall the severity of earlier symptoms. Furthermore, older women may be less sensitive to typical symptoms, and UTIs may present differently in them (Chu and Lowder, 2018; Lelie-van der Zande *et al*., 2021b). The inclusion of differential symptoms and the QoL in evaluating questionnaire results may decrease false-negative results.

A false-positive ACSS may result from typical symptoms caused by other conditions. The genitourinary syndrome of menopause includes vulvovaginal atrophy and postmenopausal modifications of the lower urinary tract (Calleja-Agius and Brincat, 2015). Blood in the urine is a typical AC symptom, but gross haematuria may also be caused by postmenopausal vaginal atrophy or an underlying malignancy (over 10% of patients) (Peterson and Reed, 2019). Smoking is associated with 25% of bladder cancers in women and patients over 35 years of age, and patients with more than a 10-pack-year smoking history have an increased risk of malignancy (Peterson and Reed, 2019). This study advises that patients with AC symptoms and gross haematuria should be referred for urologic evaluation.

In postmenopausal women, uterine prolapse may lead to voiding dysfunction symptoms such as frequent urination of small volumes and the sensation of incomplete bladder emptying after urination (Espuña Pons *et al*., 2021), possibly adding to false-positive results of typical symptoms. Typical symptoms may also result from an overactive bladder (OAB). However, UTI symptoms are generally acute, whereas OAB symptoms are generally chronic (Nik-Ahd *et al*., 2018). Differential symptoms such as vaginal or urethral discharge with typical symptoms including dysuria and abdominal pain may be caused by sexually transmitted diseases such as Chlamydia. These differential symptoms also may indicate a false-positive result of the questionnaire.

Loin pain score, mostly combined with fever, may point to tissue invasion and pyelonephritis (Johnson and Russo, 2018). The European Association of Urology (EAU) guideline advises performing urine culture and antimicrobial susceptibility testing in patients with pyelonephritis (Bonkat *et al*., 2019).

Thus, adding smoking to relevant conditions and including differential symptoms, QoL and relevant conditions in evaluating questionnaire results may decrease false-positive results.

A false-negative GP diagnosis by a urine test(s) may occur when the duration of stay of urine in the bladder is too short (NHG, 2020), possibly because postmenopausal periods are associated with increased nocturia (Tikkinen *et al*., 2008). Clinically relevant nocturia (≥ 2 voids per night) affects 2%–18% of those 20 to 40 years, rising to 28%–62% for those 70–80 years (Oelke *et al*., 2017). Nevertheless, AC increases the frequency of urinating, possibly leading to nightly urinating. Most GP practices ask for morning urine but do not automatically inform patients about sufficient length of urine stay in the bladder. Moreover, studies have found that women with typical complaints and a negative culture may still have an E. coli infection (Stamm *et al*., 1983; Hooton *et al*., 2013; Heytens *et al*., 2017). However, our study design did not allow us to detect this combination as a cause for false-negative results for urine testing in a GP practice. Also, a nitrite-positive dipstick is only found in the presence of Gram-negative uropathogens and not in the presence of enterococci, which can cause AC in a lower number of patients. Thus, the use an ACSS questionnaire with a cut-off score of 6 or higher thus might actually decrease false-negative results of urine test(s) in GP practice.

Compared with AC-positive patients, reference group women scored high for mild and moderate urinary frequency. During the cognitive assessment of the questionnaire, participants deemed the number of urinating moments low compared to the corresponding scores. Thus, increasing the number of urinating moments for mild, moderate and severe frequency may increase the distinctiveness of this typical symptom.

The EAU guideline on urological infections (Bonkat *et al*., 2021) considers the evidence for AC diagnosis in women as strong when it is based on a focused history of dysuria, frequency and urgency and absence of vaginal discharge or irritation. In this study, mean differential symptom scores of AC-positive patients for vaginal discharge were low (0.18), with scores between 0 and 2. Vaginal discharge in AC-positive patients did not differ significantly from the reference group, in alignment with an earlier study that showed that complaints of abnormal vaginal discharge were found not to decrease AC probability (Alidjanov *et al*., 2019). Thus, the absence of vaginal discharge in the EAU guideline might be re-evaluated.

The added value of the ACSS questionnaire in Dutch clinical practice compared to urine testing is that the ACSS questionnaire can be filled in easily and fast by most women and can save contact time in primary care. The questionnaire provides information on seriousness of symptoms, on differential symptoms and influence on QoL irrespective of the time of handing in urine. Moreover, relevant co-morbidities are asked out. Thus it may also improve the AC diagnosis in out-of-hours primary care (Spek *et al*., 2020). This may also be the case in other countries, as in some Canadian provinces (Beahm *et al*., 2017; 2018), New Zealand (Gauld *et al*., 2017), and Queensland (Australia) (Australia, Pharmacy Guild of., 2022a; 2022b) women with recurring UTI can consult a trained pharmacist to receive an antibiotic without a physician prescription. For AC diagnosing, these pharmacists mostly use a simple questionnaire, a urine dipstick test or a guideline for AC diagnosing. Therefore, the ACSS questionnaire may improve diagnosis of AC by community pharmacists as well.

## Strengths and weaknesses

Because of the COVID-19 pandemic, the study was interrupted but resumed after six months. Based on earlier ACSS validation studies, in the design of the study we planned to include 50 women with suspected AC from 3 different GP practices and an equal number of women in the reference group to be able to validate the questionnaire (Magyar *et al*., 2018; Di Vico *et al*., 2020). However, after including 9 patients in the first GP practice and 20 women in the reference group in the first pharmacy COVID-19 forced us to interrupt the study. In the second pharmacy, we included 50 women for the reference group over a short period and decided to include 50 patients for the patient group in the second GP practice. The characteristics of the women included at the first location were comparable to those at the second location.

A strength was that the study was performed in as well an urban as a rural setting. A weakness was that the inclusion procedure in the second GP practice had to be adapted because of the pandemic. In the first location, patients were asked in person to enrol in the study and patients filled in questionnaire A immediately after handing in their urine sample. However, in the second location, patients handed in their urine at an unattended counter where they could find information about the study and filled in a consent form with permission to be telephoned by the researcher. The researcher allowed these patients to answer the ACSS questionnaire form A, form B and the identifying characteristics telephonically. Thereby, patients at the second GP practice filled in the questionnaire up to four days after handing in the urine sample, mostly after the start of antibiotic treatment. On this account, the patient was asked about the severity of symptoms at the time of handing in their urine sample. The adapted procedure impeded the inclusion of patients and made it impossible to calculate the patient response rate. Since only patients fluent in Dutch could be included, patients with low health literacy are missing, and thus, our results may not be representative for this patient group. Primary, secondary and higher education levels for the patient group with 33%, 37% and 30%, respectively, are largely similar with national data for women 15–75 years in 2020 which were 26%, 38% and 34%, respectively (CBS, 2020). Thus, the overall education level of the patient group was in the range of the overall national education level.

This study was performed during the COVID-19 epidemic. The mean score for influence on QoL social activities was 55% lower than reported in an earlier study (Alidjanov *et al*., 2016), probably because social activities were minimal or not possible during lockdown periods. Moreover, all patients with a divergent result for the ACSS questionnaire and urine test(s) were included in the GP practice at the second location. The differences between the results of the questionnaire and urine test(s) may have been caused by the time interval between the GP practice visit and filling in the questionnaire. Because patient numbers in the first GP practice were much smaller than in the second GP practice, low statistical power prohibited statistical analysis of these differences.

## Summary and conclusion

Although the reliability of the ACSS questionnaire to establish AC was comparable to earlier studies, for use in primary care additional reduction in false-positive and false-negative results was possible, achieved by including scores for differential symptoms, QoL, relevant conditions and moderate or severe scores for blood in the urine in the evaluation of the questionnaire in patients with a typical symptom score of 6 or higher.

## Supplementary material

For supplementary material accompanying this paper visit http://doi.org/10.1017/S1463423622000627. click here to view supplementary material
