# Supplementary material for: Diagnosis of acute cystitis in primary care: symptom-based versus urinalysis-based diagnosis
Source: Prim Health Care Res Dev. 2022 Nov 17;23:e74. doi: 10.1017/S1463423622000627 (PMC9706387; doi:10.1017/S1463423622000627)
Supplement: Supplementary file 1 [file S1463423622000627sup001.pdf]

Suppl. Fig. 1A - Dutch Acute Cystitis Symptom Score (ACSS) - Part A
Vragenlijst blaasontsteking Eerste bezoek - Deel A

Vragenlijst ingevuld (Datum): (dd / mm / jjjj)

Wilt u aankruisen of u in de afgelopen 24 uur last hebt gehad van een of meer van de volgende klachten en hoe erg de klachten waren

		Geen last (0)	Een beetje (1)	Tamelijk erg (2)	Heel erg (3)
Gebruikelijke klachten	1 Vaak plassen van kleine hoeveelheden urine (vaker naar de wc)	☐ 4x of minder per dag	☐ 5-6x per dag	☐ 7-8x per dag	☐ 9x of meer per dag
	2 Plotselinge, sterke aandrang om te plassen	☐	☐	☐	☐
	3 Voelt u branderige pijn als u plast	☐	☐	☐	☐
	4 Gevoel dat u na het plassen weer zou moeten plassen (gevoel dat de blaas niet helemaal leeg is na het plassen)	☐	☐	☐	☐
	5 Pijn in de onderbuik (lager dan uw navel), ook als u niet plast	☐	☐	☐	☐
	6 Bloed in de urine gezien? (geen menstruatiebloed)	☐	☐	☐	☐

totaalscore van gebruikelijke klachten:________

Andere klachten	7 Pijn in een of beide flanken (pijn in een of beide zijkanten van onderrug)	☐	☐	☐	☐
	8 Meer of andere vaginale afscheiding dan gewoonlijk (hoeveelheid, kleur en/of geur)	☐	☐	☐	☐
	9 Afscheiding uit de plasopening zonder dat u plast	☐	☐	☐	☐
	10 Gevoel dat u koorts of verhoging heeft?	☐	☐	☐	☐
	10a Als u uw temperatuur gemeten heeft, wilt u dan aankruisen hoe hoog die was ☐ Ja · Nee	≤37,5 ˚C	☐ 37,6-37,9 ˚C	☐ 38,0-38,9 ˚C	☐ ≥39,0 ˚C

totaalscore van andere klachten:________

Kwaliteit van leven

11 Wilt u aankruisen hoeveel ongemak u de afgelopen 24 uur hebt gehad van uw klachten (kies het antwoord dat het dichtst bij komt)

0 Geen ongemak (helemaal geen klachten, ik voel me net zo als ik me gewoonlijk voel)

1 Een beetje ongemak (ik voel me een beetje slechter dan gewoonlijk)

2 Tamelijk veel ongemak (ik voel me behoorlijk slecht)

3 Heel veel ongemak (ik voel me vreselijk slecht)

12 Wilt u aankruisen hoe erg de klachten de afgelopen 24 uur uw dagelijkse activiteiten/werk hebben beïnvloed

0 Geen invloed (heb zoals gewoonlijk mijn dagelijkse activiteiten/werk kunnen uitvoeren of had niets gepland)

1 Een beetje invloed (heb mijn dagelijkse activiteiten/werk met enig ongemak kunnen uitvoeren)

2 Tamelijk veel invloed (heb mijn dagelijkse activiteiten/werk met moeite kunnen uitvoeren)

3 Heel veel invloed (bijna onmogelijk om mijn dagelijkse activiteiten/werk uit te voeren)

13 Wilt u aankruisen hoe erg de klachten de afgelopen 24 uur uw sociale activiteiten (vrienden ontmoeten, uitgaan..) hebben beïnvloed

0 Geen invloed (heb alles kunnen doen wat ik van plan was of was niets van plan*)*

1 Een beetje invloed (heb minder kunnen doen dan ik van plan was)

2 Tamelijk veel invloed (heb veel minder kunnen doen dan ik van plan was)

3 Heel veel invloed (door de klachten heb ik niets kunnen doen van wat ik van plan was)

totaalscore van kwaliteit van leven:_______

14 Wilt u aankruisen of het volgende wel of niet op u van toepassing is

Aanvullende vragen			
	Menstrueert u (heeft u maandelijkse bloedingen)?	☐ Ja	Nee
	Heeft u klachten zoals buikpijn in de week voor de menstruatie (Premenstruele klachten= PMS)?	Ja	Nee
	Heeft u last van overgangsklachten (zoals opvliegers)?	Ja	Nee
	Bent u zwanger?	Ja	Nee
	Heeft uw arts vastgesteld dat u diabetes mellitus heeft (suikerziekte)	Ja	Nee

Lelie-van der Zande et al. 2022 submitted for publication

Suppl. Fig. 1B - Dutch Acute Cystitis Symptom Score (ACSS) - Part B
Vragenlijst blaasontsteking Vervolgbezoek - Deel B

Vragenlijst ingevuld (Datum): (dd / mm / jjjj)

Wilt u aankruisen of uw klachten (symptomen) veranderd zijn sinds het invullen van de eerste vragenlijst (kies het antwoord dat het beste weergeeft hoe u zich voelt)

Dynamiek	
	0 Ja, ik voel me zoals gewoonlijk, alle klachten zijn verdwenen
	1 Ja, ik voel me veel beter, de meeste klachten zijn verdwenen,
	2 Ja, ik voel me een beetje beter, een paar klachten zijn minder geworden of verdwenen
	3 Nee, ik voel me slechter, mijn klachten zijn erger geworden

Wilt u aankruisen of u in de afgelopen 24 uur last hebt gehad van een of meer van de volgende klachten en hoe erg de klachten waren

		Geen last (0)	Een beetje (1)	Tamelijk erg (2)	Heel erg (3)
Gebruikelijke klachten	1 Vaak plassen van kleine hoeveelheden urine (vaker naar de wc)	☐ 4x of minder per dag	☐ 5-6x per dag	☐ 7-8x per dag	☐ 9x of meer per dag
	2 Plotselinge, sterke aandrang om te plassen	☐	☐	☐	☐
	3 Voelt u branderige pijn als u plast	☐	☐	☐	☐
	4 Gevoel dat u na het plassen weer zou moeten plassen (gevoel dat de blaas niet helemaal leeg is na het plassen)	☐	☐	☐	☐
	5 Pijn in de onderbuik (lager dan uw navel), ook als u niet plast	☐	☐	☐	☐
	6 Bloed in de urine gezien? (geen menstruatiebloed)	☐	☐	☐	☐

Totaalscore van gebruikelijke klachten:________

Andere klachten	7 Pijn in een of beide flanken (pijn in een of beide zijkanten van onderrug)	☐	☐	☐	☐
	8 Meer of andere vaginale afscheiding dan gewoonlijk (hoeveelheid, kleur en/of geur)	☐	☐	☐	☐
	9 Afscheiding uit de plasopening zonder dat u plast	☐	☐	☐	☐
	10 Gevoel dat u koorts of verhoging heeft?	☐	☐	☐	☐
	10a Als u uw temperatuur gemeten heeft, wilt u dan aankruisen hoe hoog die was ☐ Ja · Nee	≤37,5 ˚C	☒ 37,6-37,9 ˚C	☐ 38,0-38,9 ˚C	☐ ≥39,0 ˚C

totaalscore van andere klachten:________

Kwaliteit van leven

11 Wilt u aankruisen hoeveel ongemak u de afgelopen 24 uur hebt gehad van uw klachten (kies het antwoord dat het dichtst bij komt)

0 Geen ongemak (helemaal geen klachten, ik voel me net zo als ik me gewoonlijk voel)
1 Een beetje ongemak (ik voel me een beetje slechter dan gewoonlijk)
2 Tamelijk veel ongemak (ik voel me behoorlijk slecht)
3 Heel veel ongemak (ik voel me vreselijk slecht)

12 Wilt u aankruisen hoe erg de klachten de afgelopen 24 uur uw dagelijkse activiteiten/werk hebben beïnvloed

0 Geen invloed (heb zoals gewoonlijk mijn dagelijkse activiteiten/werk kunnen uitvoeren of had niets gepland)
1 Een beetje invloed (heb mijn dagelijkse activiteiten/werk met enig ongemak kunnen uitvoeren)
2 Tamelijk veel invloed (heb mijn dagelijkse activiteiten/werk met moeite kunnen uitvoeren)
3 Heel veel invloed (bijna onmogelijk om mijn dagelijkse activiteiten/werk uit te voeren)

13 Wilt u aankruisen hoe erg de klachten de afgelopen 24 uur uw sociale activiteiten (vrienden ontmoeten, uitgaan..) hebben beïnvloed

0 Geen invloed (heb alles kunnen doen wat ik van plan was of was niets van plan)
1 Een beetje invloed (heb minder kunnen doen dan ik van plan was)
2 Tamelijk veel invloed (heb veel minder kunnen doen dan ik van plan was)
3 Heel veel invloed (door de klachten heb ik niets kunnen doen van wat ik van plan was)

totaalscore van kwaliteit van leven:________

14 Wilt u aankruisen of het volgende wel of niet op u van toepassing is

Aanvullende vragen			
	Menstrueert u (heeft u maandelijkse bloedingen)?	Ja	Nee
	Heeft u klachten zoals buikpijn in de week voor de menstruatie (Premenstruele klachten= PMS)?	Ja	Nee
	Heeft u last van overgangsklachten (zoals opvliegers)?	Ja	Nee
	Bent u zwanger?	Ja	Nee
	Heeft uw arts vastgesteld dat u diabetes mellitus heeft (suikerziekte)	Ja	Nee

Lelie-van der Zande et al. 2022 submitted for publication
